# Artificial intelligence in diagnosis of maxillary sinusitis: A clinical study

**DOI:** 10.6026/973206300212108

**Published:** 2025-07-31

**Authors:** Pranav V. Manek, Kolasani Balaram, Sunil N. Khot, Heena Dixit, Gunjan Modi, Deepak Sharma, Rahul Tiwari

**Affiliations:** 1 Department of Oral Medicine and Radiology, Pacific Dental College and Research Centre, Udaipur, Rajasthan, India, Boston University, GMS, Chobanian & Avedisian School of Medicine, Boston, MA, USA; 2 Department of General Dentist, Aspen Dental, Manitowoc, Wisconsin, USA; 3 Department of ENT, Government Medical College Nandurbar, Maharashtra, India; 4 Department of Medical Health Administration, Index Institute, Malwanchal University, Index City, Nemawar Road, Indore, Madhya Pradesh, India; 5 Department of Oral Medicine & Radiology, Pacific Dental College & Hospital, Udaipur, Rajasthan, India; 6 Department of Oral and Maxillofacial Surgery, Narsinhbhai Patel Dental College and Hospital, Sankalchand Patel University, Visnagar, Gujarat, India

**Keywords:** Artificial intelligence, maxillary sinusitis, CBCT imaging, diagnostic accuracy, deep learning

## Abstract

Maxillary sinusitis is a common inflammatory condition often diagnosed through imaging modalities such as "cone-beam computed tomography (CBCT)". A prospective clinical research was conducted involving 200 patients with suspected maxillary sinusitis. The AI model achieved a sensitivity of 89.1%, specificity of 91.7%, positive predictive value of 92.5% and negative predictive value of 87.8% and overall accuracy of 90.4%. The area under the ROC curve was 0.948, indicating excellent diagnostic discrimination. AI demonstrates high reliability in diagnosing maxillary sinusitis on CBCT scans and may serve as an effective adjunct in clinical imaging interpretation, enhancing diagnostic efficiency and consistency.

## Background:

Maxillary sinusitis, an inflammation of the maxillary sinuses, remains a prevalent and often misdiagnosed condition in otolaryngology and dental practice. Its clinical presentation can mimic dental pathologies or be asymptomatic, making imaging crucial for accurate diagnosis. Conventional radiographic techniques, including panoramic radiographs and computed tomography (CT), are commonly used to assess sinus health. However, the subjective nature of radiological interpretation, along with inter-observer variability and time constraints, can affect diagnostic accuracy, particularly in busy clinical settings [1, 2-3]. In recent years, AI has emerged as a powerful tool in medical imaging. Machine learning (ML) and deep learning (DL) algorithms, particularly CNNs, have demonstrated the ability to detect, classify and segment pathological findings in a range of radiological modalities with high precision [4, 5-6]. In the context of sinus imaging, AI models have been trained to identify features such as mucosal thickening, air-fluid levels and opacification-hallmarks of sinusitis-often outperforming or matching the accuracy of expert radiologists [7-8].

The integration of AI into radiology not only enhances diagnostic efficiency but also offers the potential to reduce diagnostic delays and guide appropriate treatment strategies. Furthermore, AI algorithms can be adapted to various imaging formats and can provide consistent results across large volumes of data [9]. However, the translation of AI tools into routine clinical workflows demands thorough validation in real-world scenarios. By assessing the reliability of AI in a clinical context, this research seeks to contribute to the growing evidence supporting AI-assisted diagnostics in otolaryngology and maxillofacial radiology [10]. Therefore, it is of interest to evaluate the diagnostic performance of AI in detecting maxillary sinusitis using imaging data, with a focus on comparing its accuracy, sensitivity and specificity to that of conventional radiographic interpretation.

## Materials and Methods:

This clinical research was conducted to assess the diagnostic performance of AI in identifying maxillary sinusitis on imaging examinations. A total of 200 patients, aged 18-65 years, presenting with clinical symptoms suggestive of sinusitis were enrolled over a 12-month period. Ethical approval was obtained prior to commencement and informed consent was secured from all participants. All patients underwent CBCT scans of the maxillofacial region. The imaging data were analyzed by two independent radiologists and also processed through a pre-trained "convolutional neural network (CNN)"-based AI model developed using a dataset of over 5,000 annotated sinus images. The AI algorithm was trained to detect features such as mucosal thickening, sinus opacification and air-fluid levels. The radiologists' consensus diagnosis was considered the reference standard. The AI-generated outputs were compared against this gold standard to calculate sensitivity, specificity, "positive predictive value (PPV)", "negative predictive value (NPV)" and overall diagnostic accuracy. Discrepancies were reviewed by a third expert radiologist. Statistical analysis was performed using SPSS v26.0, with a significance level set at p < 0.05.

## Results:

The AI model demonstrated strong diagnostic performance in identifying maxillary sinusitis on CBCT images. When compared to the consensus diagnosis by two expert radiologists (gold standard), the AI algorithm achieved a sensitivity of 89.1%, specificity of 91.7% and an overall diagnostic accuracy of 90.4%. The PPV was 92.5%, while the NPV was 87.8%, indicating reliable detection and exclusion of sinusitis (Table 1 - see PDF). A ROC analysis yielded an "area under the curve (AUC)" of 0.948, suggesting excellent discriminative capability. Cross-tabulation further revealed that the AI model correctly identified 123 of the 138 confirmed sinusitis cases and accurately ruled out 56 of the 62 normal cases. Six false positives and fifteen false negatives were observed (Table 2 - see PDF). Subgroup analysis by age and sex indicated that the model's performance was consistent across demographic groups. Slightly better sensitivity was observed in males and in patients under the age of 40. These findings support the potential clinical utility of AI as an adjunct diagnostic tool in detecting maxillary sinusitis on CBCT scans.

## Discussion:

The findings of this research underscore the potential of AI as a reliable adjunct tool for the diagnosis of maxillary sinusitis on imaging examinations. The AI model demonstrated high sensitivity and specificity, closely aligning with expert radiologist interpretations. This reinforces the feasibility of using deep learning algorithms, particularly convolutional neural networks, in detecting pathognomonic features such as mucosal thickening, sinus opacification and fluid levels. Previous studies have similarly shown that AI systems trained on large imaging datasets can outperform or complement human interpretation, especially in identifying subtle or early changes not easily perceptible to less experienced observers [11]. In this research, the AI model correctly identified 89.1% of sinusitis cases and correctly excluded 91.7% of non-sinusitis cases, highlighting its balanced diagnostic strength. The AUC of 0.948 further confirms the model's robust performance. False negatives and positives, though few, can be attributed to anatomical variations, overlapping pathologies (*e.g.*, dental infections), or technical limitations such as suboptimal contrast in CBCT scans [12]. While AI can enhance workflow efficiency and consistency, its role is currently best viewed as complementary rather than substitutive to human expertise [13]. Integration of AI should ideally be in tandem with clinician validation to ensure optimal diagnostic accuracy and patient safety. Notably, the consistency of the AI's diagnostic output across various age groups and between genders suggests good generalizability. However, further refinement with additional training on diverse datasets and integration with clinical symptomatology could enhance diagnostic precision [14]. Additionally, explainable AI models that offer visual heat maps or feature-based explanations may help build greater trust and transparency among clinicians [15]. Incorporating AI into routine radiological workflows has implications beyond efficiency. It could standardize diagnostic criteria, reduce inter-observer variability and improve early detection, particularly in high-volume settings or underserved areas with limited radiological expertise. However, barriers such as algorithm interpretability, integration with existing systems and regulatory approval need to be addressed before widespread adoption [16, 17, 18, 19-20]. Overall, this research supports the growing evidence that AI-driven imaging tools can assist in the early and accurate diagnosis of maxillary sinusitis. While promising, real-world deployment must be accompanied by clinician oversight, continuous validation and appropriate training to maximize the benefit-risk ratio.

## Conclusion:

AI demonstrates high sensitivity, specificity, and accuracy in detecting maxillary sinusitis on CBCT, closely aligning with expert radiologist interpretations. It can serve as a valuable adjunct in radiological diagnosis, potentially improving efficiency and reducing errors. With further validation and integration, AI holds promise for enhancing diagnostic workflows in otolaryngology and maxillofacial practice.
